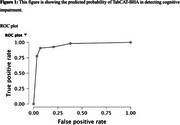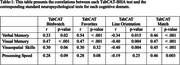# TabCAT Brain Health Assessment for Detecting Cognitive Impairment in Israeli Arabs. Discriminant and Concurrent Validity with Standard Tools

**DOI:** 10.1002/alz70857_100951

**Published:** 2025-12-25

**Authors:** Rafi Haddad, Elena Tsoy, Judith Aharon‐Peretz, Tali Fisher, David Tanne, Rawan Ayoub, Yarovinsky Natalya, Rachel Ben‐Hayun, Victor Valcour, Katherine L. Possin

**Affiliations:** ^1^ Global Brain Health Institute, University of California, San Francisco, San Francisco, CA, USA; ^2^ Rambam Health Care Campus, Haifa, Israel; ^3^ Memory and Aging Center, University of California San Francisco, San Francisco, CA, USA; ^4^ Technion‐Israel Institute of Technology, Haifa, Israel; ^5^ Global Brain Health Institute (GBHI), University of California San Francisco (UCSF); & Trinity College Dublin, San Francisco, CA, USA; ^6^ Memory and Aging Center, Weill Institute for Neurosciences, University of California, San Francisco, San Francisco, CA, USA

## Abstract

**Background:**

Dementia poses a significant public health challenge in the Middle East and North Africa, particularly among Arabic‐speaking populations. In Israel, where Arabs constitute 21% of the population, disparities in cognitive healthcare persist partially due to the lack of culturally and linguistically adapted assessments, including brief, non‐language‐dependent measures. This study evaluated the concurrent validity and discrimination accuracy of the Tablet‐based Cognitive Assessment Tool Brain Health Assessment (TabCAT‐BHA) in Arabic‐speaking Israelis.

**Methods:**

Participants included 57 older adults with cognitive impairment (age: 67±7; 56% female; education: 13±5), comprising 36 with Mild Cognitive Impairment (MCI) and 21 with dementia, and 39 cognitively normal individuals (age:62±5; 54% female; education: 15±5). Diagnoses were based on clinical criteria, and all cognitively impaired older adults completed standard locally validated paper‐and‐pencil tests: CVLT‐Short Form delayed recall (verbal memory), Benson Complex Figure delayed recall (visual memory) and copy (visuospatial skills), and Trails A (processing speed). Z‐scores were derived from established norms for Hebrew‐speaking individuals. All participants completed the Arabic version of the TabCAT‐BHA, including Birdwatch (visual associative memory), Favorites (verbal‐visual associative memory), Match (executive function), and Line Orientation (visuospatial skills). Discrimination accuracy was assessed with logistic regressions and ROC curves, adjusting for age, sex, and education. Pearson's correlations were used to assess concurrent validity with the standard tests.

**Results:**

The TabCAT‐BHA demonstrated excellent discrimination accuracy (AUC = .97, sensitivity = .93, specificity = .79) for detecting cognitive impairment (Figure 1). Strongest correlations were observed between TabCAT Birdwatch with visual memory (*r* = .47, *p* < .001); Favorites with verbal memory (*r* = .54, *p* < .001); and Line Orientation (reverse scored) with visuospatial skills (*r* = ‐0.40, P = .009; Table 1). Match was moderately associated with all examined domains (*r* = .45‐.47, all *p* ≤ .003; Table 1), which is unsurprising given a well‐documented role of executive functions in cognitive performance across domains.

**Conclusions:**

The TabCAT‐BHA is a valid and efficient tool for detecting cognitive impairment in Arabic‐speaking Israelis. Future studies will refine norms and examine performance only among those with MCI to confirm utility for early disease.